# Transient synaptic refinement and glial adaptations after whisker denervation in adult mice

**DOI:** 10.3389/fncel.2026.1941129

**Published:** 2026-09-30

**Authors:** Sean Collier, Laxmi Iyer, Kimberly R. Byrnes, Emily Petrus

**Affiliations:** 1 Neuroscience Graduate Program, Uniformed Services University of the Health Sciences, Bethesda, MD, United States; 2 Henry M. Jackson Foundation for the Advancement of Military Medicine, Bethesda, MD, United States; 3 Department of Anatomy, Physiology and Genetics, Uniformed Services University of the Health Sciences, Bethesda, MD, United States

**Keywords:** adult plasticity, injury adaptations, microglial morphology, sex-specific neural adaptations, thalamocortical plasticity

## Abstract

Loss of sensation after injury causes profound changes in neural circuits, impacting sensory perception and recovery. Overcoming sensory loss requires neural plasticity to support accurate processing of remaining sensory afferents, but the mechanisms by which circuit plasticity occurs in adults are not well understood. Despite decades of studies manipulating the mouse whisker sensory system, the impact of sex, glial activity, and time since injury has not yet been clarified. We performed unilateral whisker denervation in adult male and female mice and characterized synaptic and glial responses at multiple time points after injury. We determined that whisker denervation leads to a transient loss of thalamocortical and intracortical putative excitatory synapses in the deprived sensory cortex. This loss corresponds with a transient shift in microglial morphology, with increased proportions of ramified-shaped microglia and decreased proportions of amoeboid-shaped microglia. Most of these results were more robust in female mice, highlighting the importance of powering studies for sex-specific adaptations. Importantly, these results identify temporally restricted circuit plasticity that should be considered when implementing post-injury interventions to support recovery.

## Highlights

- Whisker denervation leads to a transient loss of putative excitatory synapses in the sensory cortex of adult mice.
- Microglia morphology decreases amoeboid, but increases ramified phenotypes 7 days after whisker denervation.
- Putative synapse and microglial adaptations return to sham levels by 14 days post-sensory loss.
- Female mice may have a more robust response to sensory loss than male mice.

## 1 Introduction

The brain is comprised of a symphony of neurons and glia, which together form circuitry to interpret incoming sensory signals. Neural connections receive afferent input from the periphery, which is routed through the thalamus into the cortex, where first- and higher-order sensory processing occurs (Douglas and Martin, 2004; Staiger and Petersen, 2021). These thalamocortical (TC) synaptic connections are maintained and sculpted by glia in juvenile and adult mammalian systems in complex ways (Erzurumlu and Gaspar, 2012; Faust et al., 2021).

Modulating whisker sensation in mice has provided valuable insights into experience-dependent synaptic and functional adaptations in developing and adult circuitry. Whiskers on the face are homotopically represented in layer 4 of the primary somatosensory barrel cortex (S1BC), such that a round, barrel-shaped group of cells receives input from the TC connection corresponding to only one whisker (Staiger and Petersen, 2021). Somatosensory TC connections are initially highly plastic in rodents (Erzurumlu and Gaspar, 2012), but these connections become resistant to change after the first postnatal week. Ablating a unilateral whisker set at postnatal day 4 in mice reduces TC synapse density within a week (Faust et al., 2025; Gunner et al., 2019). Adult whisker trimming yields mixed results, with some reporting reduced TC density in deprived barrels (Oberlaender et al., 2012; Wimmer et al., 2010), while others find TC patterning remains in place (Woolsey and Wann, 1976) with unchanged whisker response characteristics (Fox, 1992).

Microglia, the resident immune cells of the brain, play a pivotal role in the wiring of sensory systems of juvenile, pre-critical period mice. Microglia surveil developing neural connections in the brain and readily phagocytose underused synapses, leaving active connections untouched (Andoh et al., 2020; Hong et al., 2016). However, their role in adult circuit remodeling after changes in experience is debated. For example, although microglia are required for visual TC synaptic refinement after monocular inactivation (Schafer et al., 2012), their absence has no effect on ocular dominance plasticity (Brown et al., 2024). What might account for these varying results? Three important, potentially intersecting biological factors may influence adult circuit adaptations: temporal dynamics, the type of synapses studied, and sex as a biological variable.

We address these three issues by using a mouse model of unilateral whisker denervation, which is achieved by transecting the infraorbital nerve (IONX). This model has a well-described timeline for intact S1BC TC synaptic plasticity (Chung et al., 2017; Iyer et al., 2026; Jie et al., 2025; Petrus et al., 2019; Yu et al., 2012), but glial adaptations and neural activity on the deprived S1BC are less well understood. We performed unilateral whisker denervation or sham surgery in adult mice and examined TC and intracortical (IC) innervation density, neuronal activity, and microglial morphology at various time points in male and female cohorts. We found a transient reduction in TC terminal labeling, reduced spontaneous neural activity, and a shift toward ramified microglial morphology 7 days after whisker denervation. Surprisingly, all measurements returned to sham levels by 14–28 days after IONX. These results indicate that the timing after injury and sex-specific synaptic and glial adaptations to sensory loss play important roles in denervation-induced circuit remodeling. These temporal and sex-specific changes may explain the variable results from previous sensory loss studies, which have historically been performed in male mice or insufficiently powered cohorts to detect sex-specific changes. Because circuit remodeling is proposed to underlie chronic pain or poor recovery after injury (Flor et al., 2006; Woolf and Salter, 2000), this may be a mechanism by which females plural have higher rates of discomfort after denervation or limb loss injury (Mioton et al., 2020).

## 2 Methods

### 2.1 Animals

All procedures were approved by the Uniformed Services University of the Health Sciences Institutional Animal Care and Use Committee (IACUC) under protocol APG-25-081. Facilities are accredited by the Association for Assessment and Accreditation of Laboratory Animal Care (AAALAC). Five-week-old male and female C57BL/6J mice (Strain#000664, RRID: IMSR_JAX:000664) were obtained from Jackson Laboratories (Bar Harbor, ME) and allowed to habituate to the animal facility for 1 week before experimental intervention. Littermates were housed 2–4 per cage with food and water ad libitum on a 12-h light/dark cycle.

### 2.2 Infraorbital nerve transection surgery (IONX)

All surgeries were performed under aseptic techniques. Six-week-old mice were anesthetized with an intraperitoneal (IP) injection of a ketamine/xylazine/saline cocktail (80 mg/kg ketamine and 10 mg/kg xylazine). After mice were unresponsive to paw pinch, the right side of the face was shaved, and an incision caudal to the whisker pad was made to visualize the infraorbital nerve (ION) bundle. For sham mice, the procedure was concluded at this step, with the incision closed with a single suture and tissue glue (TissueMend II, Veterinary Product Laboratories). In IONX mice, the ION bundle was cut with scissors, and the incision was closed as described above. Animals then received an IP injection of Antisedan (1 mg/kg; Atipamezole, Zoetis) to reverse the anesthesia and subcutaneous Meloxicam extended release (4 mg/kg). Animals remained on a heated surface and returned to the animal facility after they were fully ambulatory. Denervation was confirmed by inspection of the infraorbital nerve bundle during euthanasia. Regrowth was not observed, consistent with our previous findings (Petrus et al., 2019).

### 2.3 Immunohistochemistry

Seven, 14 or 28 days after Sham/IONX surgery, mice were anesthetized with 5% isoflurane vapors and transcardially perfused with PBS, followed by 4% PFA. Brains were removed and successively fixed for 24 h in each solution at 4 °C: 4% paraformaldehyde (PFA), followed by 15 and 30% sucrose in Phosphate-buffered saline (PBS), and then cryoprotected in Optimal Cutting Temperature compound at −80 °C. Coronal sections (30-μm thick) containing S1BC were collected using a cryostat (Leica Biosystems (Deerpark, IL) CM1950). Free-floating sections (four per animal) were washed for 10 min in PBS. Sections for Iba1/CD68 analysis were blocked in 10% NGS and 0.3% Triton-X100 in PBS (NGST) for 90 min. Sections for VGlut1/VGlut2/PSD95 analysis were blocked in 0.1% Tween and 0.01 g/mL freshwater fish skin in NGST for 90 min. Sections were incubated for 48 h at 4 °C in primary antibodies diluted in NGST. Antibodies include rabbit-anti Iba1 (1:1000, 019–19,741 Wako (Osaka, Japan)), rat-anti CD68 (1:2000, 14–0681-82, Thermo Fisher (Waltham, MA)), guinea pig-anti VGlut2 (1:2000, AB2251-I, Millipore Sigma (St. Louis MO)), guinea pig anti-VGlut1 (1:2000, AB5905, MilliporeSigma), and PSD 95 (1:300, 51–6,900, Thermo Fisher). After washing three times in rabbit-anti-PBS, sections were incubated in NGST for 2 h at room temperature, protected from light in secondary antibodies: Iba1/CD68: goat-anti rabbit 488 (1:1000, A-11008, Thermo Fisher) and goat-anti rat 594 (1:2000, A48264, Invitrogen (Carlsbad, CA)); VGlut2/PSD95: goat-anti guinea pig 488 (1:2500, ab150185, Abcam (Cambridge, UK)) and goat-anti rabbit 594 (1:1000, A11072, Invitrogen) in NGST for 2 h at room temperature. After three washes, sections were incubated with DAPI (1:1000). Sections were mounted with Prolong Gold mounting medium, and coverslip edges were sealed with clear nail polish.

### 2.4 Imaging

Iba1/CD68 images were captured on a Zeiss AxioScan. Z1 microscope at 20x. Tiled, 20 μm Z-stack images for each cortex were collected at 0.5 μm or 1 μm steps for DAPI (350 nm), Iba1 (488 nm), and CD68 (546 nm). Extended depth of field (EDF) processing was used to generate a single plane image of the brightest points of each Z-stack. ROIs were drawn in S1BC of the deprived S1BC using a mouse brain atlas (Paxinos & Franklin). Layer 4 (L4) was identified as the dense band of DAPI nuclear staining approximately 400 μm from the pia. Two L4 ROIs in the Iba1 channel were selected from the deprived S1BC for each section, and 3–6 sections were collected per animal. Images for VGlut2 analysis were captured on a Nikon Ti2-E inverted microscope at 20x. Tiled, full slice images were taken for DAPI and VGlut2. Six to eight sections were processed for each animal, each containing two ROIs within L4. Data from these slices were averaged into one value per mouse (n).

#### 2.4.1 Putative synapse image aquisition

High-resolution z-stacks of S1BC L4 were acquired using a Zeiss LSM 980 confocal microscope with a 63x oil-immersion objective (NA 1.4). For each section, 2–3 image stacks were acquired from L4. Z-stacks consisted of 30 optical sections captured at a step size of 0.17 μm (total depth ≈ 5 μm), with a resolution of 1,024 × 1,024 pixels and 1.64x digital zoom.

### 2.5 Electrophysiological recordings

#### 2.5.1 Slice preparation

Seven days after sham/IONX surgeries, acute slices were made for whole-cell electrophysiological recordings. Mice were anesthetized using isoflurane vapors until the absence of the righting reflex was observed. Mice were transcardially perfused with ice-cold dissection buffer (80 mM NaCl, 3.5 mM KCl, 1.25 mM H_2_PO_4_, 25 mM NaHCO_3_, 4.5 mM MgSO_4_, 0.5 mM CaCl_2_, 10 mM glucose, and 90 mM sucrose), which was bubbled continuously with a 95% O_2_/5% CO_2_ gas mixture. Brain blocks containing S1BC were dissected and coronally sectioned into 300-μm-thick slices using a Leica VT1000S vibratome (Leica Biosystems Inc.). Slices were incubated for 60 min at room temperature before recordings began.

#### 2.5.2 Recordings

Slices were transferred to a submersion-style recording chamber mounted on a fixed stage (Sutter Instruments (Novato, CA)) with an upright Nikon Eclipse FN1 microscope (Nikon Instruments (Melville, NY)) and illuminated with oblique infrared (IR) illumination. Recordings were performed in artificial cerebrospinal fluid (ACSF; 130 mM NaCl, 3.5 mM KCl, 1.25 mM NaH_2_PO_4_·H_2_O, 24 mM NaHCO_3_, 10 mM glucose, 2.5 mM CaCl_2_, and 1.5 mM MgCl_2_) bubbled with 95% O_2_/5% CO_2_ at 30 °C. The ACSF was perfused at a rate of 2 mL/min. Voltage clamp experiments used Cs-gluconate internal solution, containing the following: 130 mM Cs-gluconate, 8 mM KCl, 1 mM EGTA, 10 mM HEPES, 4 mM ATP, 5 mM QX-314; pH 7.3, 285–295 mOsm. Neurons in L4 were targeted for patch clamp recordings. Cells with an access resistance higher than 30 MΩ and input resistance lower than 100 MΩ or more than a 20% fluctuation in these values were discarded. Three cells per animal, one cell per slice, were recorded for each dataset.

#### 2.5.3 Miniature excitatory postsynaptic currents (mEPSCs) and miniature inhibitory postsynaptic currents (mIPSCs)

ACSF contained 1 μM tetrodotoxin (TTX) and 100 μM DL-2-amino-5-phosphonopentanoic acid (DL-APV). Neurons in L4 were targeted for voltage clamp recording, and mEPSCs were recorded for 3–5 min at −70 mV holding potential to isolate AMPA receptor-mediated events. After mEPSCs were recorded, the holding potential was switched to +10 mV to isolate miniature inhibitory postsynaptic currents (mIPSCs), GABAergic events. After mIPSC recordings were concluded, 20 μM Bicuculline and 10 μM NBQX were added to the ACSF. Following a 5- to 10-min wash-in period, recordings were repeated at −70 mV and +10 mV. The absence of mEPSCs and mIPSCs at their respective holding potentials indicated that these events were mediated primarily by AMPA and GABA receptor activity (Sakimoto et al., 2019). Four male sham, three female sham, four male IONX, and four female IONX mice were used to obtain 12, 9, 12, and 12 cells, respectively.

### 2.6 Analysis

#### 2.6.1 VGlut2

ROIs from the deprived S1BC were processed using an automated Python workflow. The average intensity of pixels in the 97th quantile and above was taken as the fluorescence intensity signal of VGlut2^+^ TC terminals in L4 barrels. The average intensity of pixels in the 20th to 40th quantiles was taken as the background fluorescence and non-L4 fluorescence intensity of TC labeling in the tissue. The barrel intensity of each slice was normalized to its background intensity to produce a normalized VGlut2 intensity score per slice; all normalized slices were averaged to produce animal values. Due to variation in immunohistochemical staining runs, each cohort was normalized to its average sham value; then, cohorts were combined for treatment comparisons.

#### 2.6.2 Electrophysiology

An Axon patch-clamp amplifier 700B (Molecular Devices, Sunnyvale, CA, USA) was used for patch-clamp recordings. Data were acquired through pClamp11 and analyzed with Clampfit 11.2 software (Molecular Devices, Sunnyvale, CA, USA). Then, 200 consecutive mEPSCs and mIPSCs were selected for analysis using MiniAnalysis software (Synaptosoft/BlueCell, Anyang, Korea). Statistics were performed using unpaired Student’s *t*-test between the sham and IONX groups. The values are depicted as mean +/− SEM.

#### 2.6.3 Putative VGlut1 synapse quantification

Immunolabeled puncta from presynaptic and postsynaptic components were quantified using SynBot (Image J/FIJI) (Savage et al., 2024). Maximum projections of six consecutive optical sections (corresponding with 1 μm total depth) were generated from the original z stack. Non-overlapping ROIs from three sections per mouse were analyzed. The analysis was performed per SynBot protocol,^1^ which has been validated in various tissues and appropriate controls. Two-channel localization along with the Pixel-overlap analysis type was performed for VGlut1-PSD95. Average puncta count and colocalized putative synapse numbers were compared between the sham and IONX groups using unpaired two-tailed Student’s *t*-tests.

#### 2.6.4 Microglia morphology

Microglia morphology analysis was conducted using the MicrogliaMorphology ImageJ/FIJI plugin (Kim et al., 2024). ROIs were thresholded using the Niblack local auto-threshold method at a range of 30 pixels. Single-cell images were collected if cells were between 100 and 350 pixels in size. Images were manually curated, and cells were removed from analysis if processing artifacts were detected. After curation, cells were skeletonized, and fractal analysis was performed using the FracLac plugin. FracLac and skeleton analysis outputs were collected and analyzed in R using the MicrogliaMorphology R package. Cells were identified by sample, time point, sex, and treatment. Principal component (PC) analysis was conducted, and three PCs, capturing 82.3% of the variance, were chosen to perform fuzzy k-means clustering. A k of 4 was chosen based on the silhouette method. Cluster names from Kim et al. were used based on the similarity of all clusters using a feature heatmap. Cluster proportion was calculated using only those cells that scored above 0.5 confidence for cluster assignment. Colorized images were generated using the ColorByCluster module of MicrogliaMorphology.

### 2.7 Statistics and rigor

Statistical tests were run in GraphPad Prism 10. Student’s *t*-tests and Mann–Whitney tests were used to compare sham vs. IONX mice, where *p* < 0.05 was considered statistically significant. A three-way analysis of variance (ANOVA) was used to assess the effects of treatment, time point, and sex and their interactions. A subsequent 2 way ANOVA was used to identify the 7 day timepoint as significant. A two-way multivariate analysis of variance (MANOVA) was used to assess changes in microglial morphology composition. Animal allocation, histology, imaging, and analysis were performed by an individual blinded to the experimental condition of the animals. No sections or animals were excluded from analysis. Electrophysiology experiments were analyzed by an individual blinded to the animal condition. No cells or animals were excluded from analysis.

## 3 Results

### 3.1 Rapid thalamocortical reduction after whisker denervation

We hypothesized that IONX in adults would cause a reduction in afferent thalamocortical (TC) synapses, as seen in studies of juvenile and adult mice (Gunner et al., 2019; Oberlaender et al., 2012; Wimmer et al., 2010). TC synapses express vesicular glutamate transporter 2 (VGlut2), while intracortical synapses express vesicular glutamate transporter 1 (VGlut1) (Fremeau et al., 2001). Five-week-old male and female C57BL/6J mice underwent unilateral sham or IONX surgery, which permanently removes afferent sensory drive from a complete set of whiskers on one side of the face. Seven days later, VGlut2-positive (VGlut2^+^) TC L4 “barrels” were immunolabeled with VGlut2, and the fluorescence intensity of these labeled terminals was quantified in the deprived S1BC, contralateral to whisker denervation (Figure 1A). TC afferents are arranged into barrel-like structures in L4 of S1BC (Petersen, 2007), which we identified with VGlut2^+^ immunohistochemistry. An automated Python analysis pipeline was used to isolate VGlut2^+^ L4 barrels from the background tissue and non-L4 VGlut2 staining in deeper layers (Figure 1B) (Jensen and Killackey, 1987; Wimmer et al., 2010). The fluorescence intensity of the immunolabeled VGlut2^+^ terminals was normalized to the background fluorescence and quantified. Seven days after unilateral IONX, the fluorescence intensity of VGlut2^+^ TC terminals was significantly reduced (Supplementary Table 1, *t*-test *p* = 0.003) in the deprived S1BC (Figure 1C). Interestingly, although VGlut2 fluorescence intensity did not differ between sham male and sham female mice (Supplementary Table 2, *t*-test *p* = 0.791), the IONX-induced decrease in VGlut2 fluorescence intensity was significant in female mice (*t*-test *p* = 0.005) but not in male mice (Supplementary Table 3, *t*-test *p* = 0.282) (Supplementary Figures 1A,B). A two-way ANOVA revealed no significant interaction between sex and treatment (*p* = 0.586, Supplementary Table 4). It should be noted that the male sample size (*n* = 6, 4) was smaller than the female sample size (*n* = 8, 8), limiting statistical power in the male mice. These data indicate that IONX in adult mice quickly leads to reduced TC afferents, with this adaptation appearing to be more pronounced in female than male mice.

**Figure 1:**
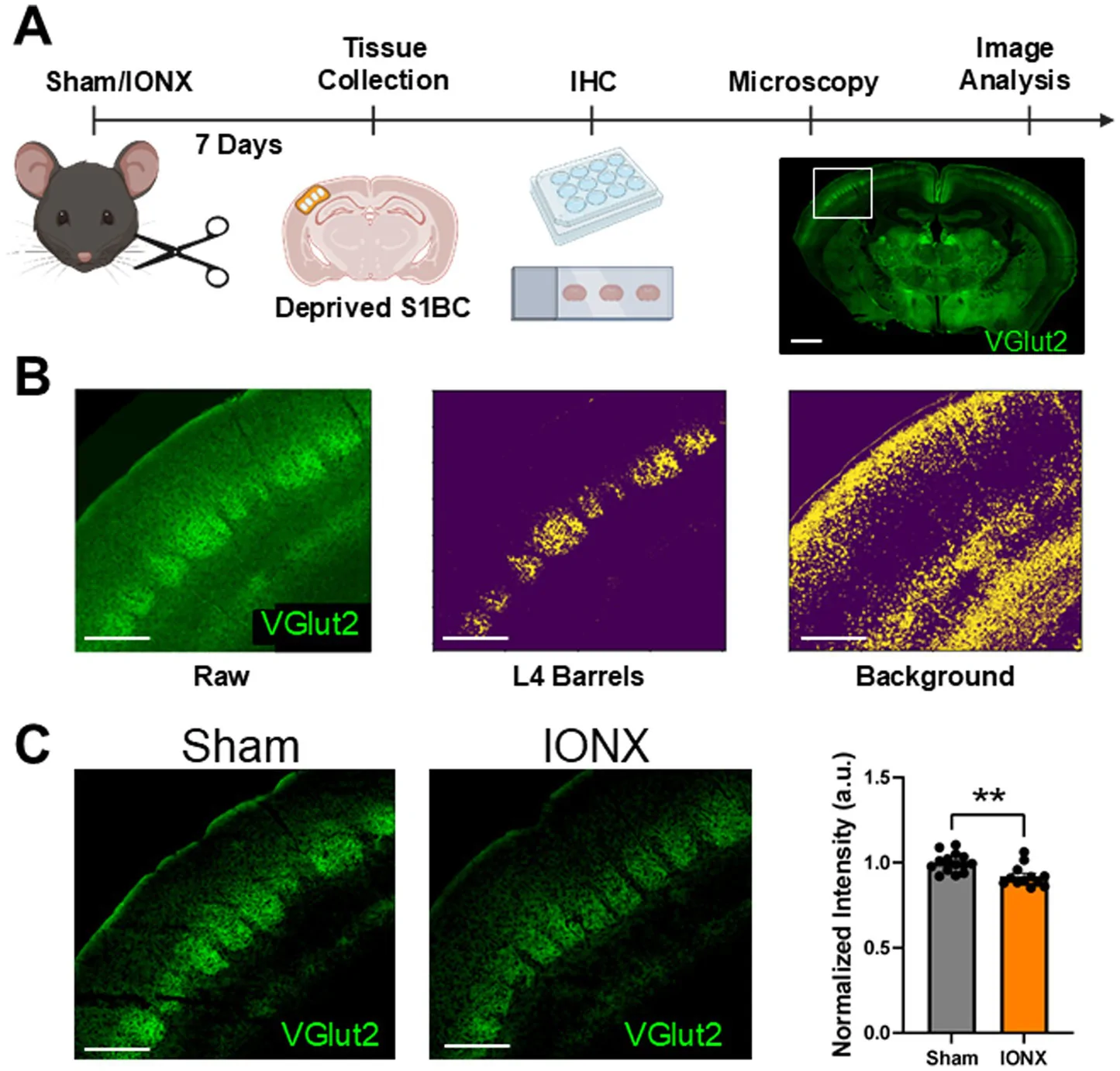
Rapid reduction in thalamocortical (TC) fluorescence intensity after whisker denervation. **(A)** Experimental timeline; immunohistochemistry (IHC) was performed 7 days after sham or infraorbital nerve transection (IONX). **(B)** Thalamocortical VGlut2-positive (VGlut2^+^) terminals are identified in layer 4 (L4) of primary somatosensory barrel cortex (S1BC, left). An automated pipeline was used to isolate fluorescence intensity of the barrels in L4 (center) compared to the background and non-L4 TC inputs to S1BC (right). **(C)** The fluorescence intensity in arbitrary units (a.u.) of VGlut2^+^ terminals is decreased in IONX mice compared to shams. Data are presented as mean ± standard error of the mean (SEM). *n* (mice) = 14 sham, 12 IONX. *T*-test. **p < 0.01. Scale bars: **A** = 1 mm; **B** and **C** = 200 μm.

### 3.2 Whisker denervation globally reduces synapse activity and numbers

IONX quickly reduced VGlut2^+^ TC terminal labeling, which may occur in response to the loss of afferent sensory-driven input to S1BC. This loss may also reduce excitatory activity in L4 neurons. Whole-cell patch clamp electrophysiology measured spontaneous excitatory and inhibitory activity in male and female mice 7 days after sham or IONX. L4 neurons were visually identified by their location within a barrel (Figure 2A), and miniature excitatory or inhibitory postsynaptic currents (mEPSCs/mIPSCs) were recorded at −70 mV and +10 mV holding potentials, respectively, in the presence of TTX and APV to block action potential-mediated synaptic activity and NMDA receptors, respectively. Recordings were repeated at both holding potentials after washing in drugs to block AMPA (NBQX) and GABA (bicuculline) receptors, to verify that mEPSCs and mIPSCs were mediated by these receptors (Figure 2B). We detected reduced mEPSC frequency in IONX mice (*t*-test, *p* = 0.006, Figure 2C, left; Supplementary Table 5) and a larger inter-event interval (Mann–Whitney test, *p* = 0.0006, Figure 2C, center). mEPSC amplitudes were unchanged after IONX, indicating that these changes were primarily presynaptic in nature (*t*-test, *p* = 0.384, Figure 2C, right; Supplementary Table 5). Next, because the balance of excitatory and inhibitory synaptic transmission is an important part of circuit remodeling, we quantified the frequency and amplitude of mIPSCs in mice after sham or IONX. We did not find a statistically significant difference in mIPSC frequency or amplitude in IONX mice compared to shams (*t*-test, *p* = 0.526, *p* = 0.574, respectively, Figure 2D; Supplementary Table 6). These results indicate that the loss of whisker-mediated afferent sensation causes a selective reduction in excitatory events 7 days after IONX. The reduced mEPSC frequency could also reflect a loss of intracortical (IC) synapses. To address this possibility, we quantified putative IC synapses by labeling their presynaptic components with VGlut1 and colocalizing their expression with postsynaptic densities (PSD95) in L4 neurons (Figure 2E). We detected decreased numbers of VGlut1^+^, PSD95, and colocalized putative synapses (*t*-test, *p* = 0.0002, *p* = 0.0001, *p* = 0.0117, respectively) in IONX compared to sham mice 7 days after IONX (Figure 2F). Although female and male sham mice were not statistically different in mEPSC/mIPSC (Supplementary Table 5, 6) or IC putative synapse (Supplementary Table 7) measurements, we again found a more robust reduction in these findings in female IONX mice than male mice. Female mEPSC frequency was significantly reduced, while male mEPSC frequency only trended down (*t*-test *p* = 0.041, 0.082, respectively, Supplementary Figures 2A,B). When split by sex, mIPSC amplitude and frequency were unchanged in male or female mice (Supplementary Figure 2C). Similarly, female IC putative synapse loss after IONX was more pronounced than that observed in male mice (Supplementary Table 7; Supplementary Figure 2D). Together, these results demonstrate that 7 days after IONX, mice lose presynaptic excitatory partners, comprised of both TC and IC connections.

**Figure 2:**
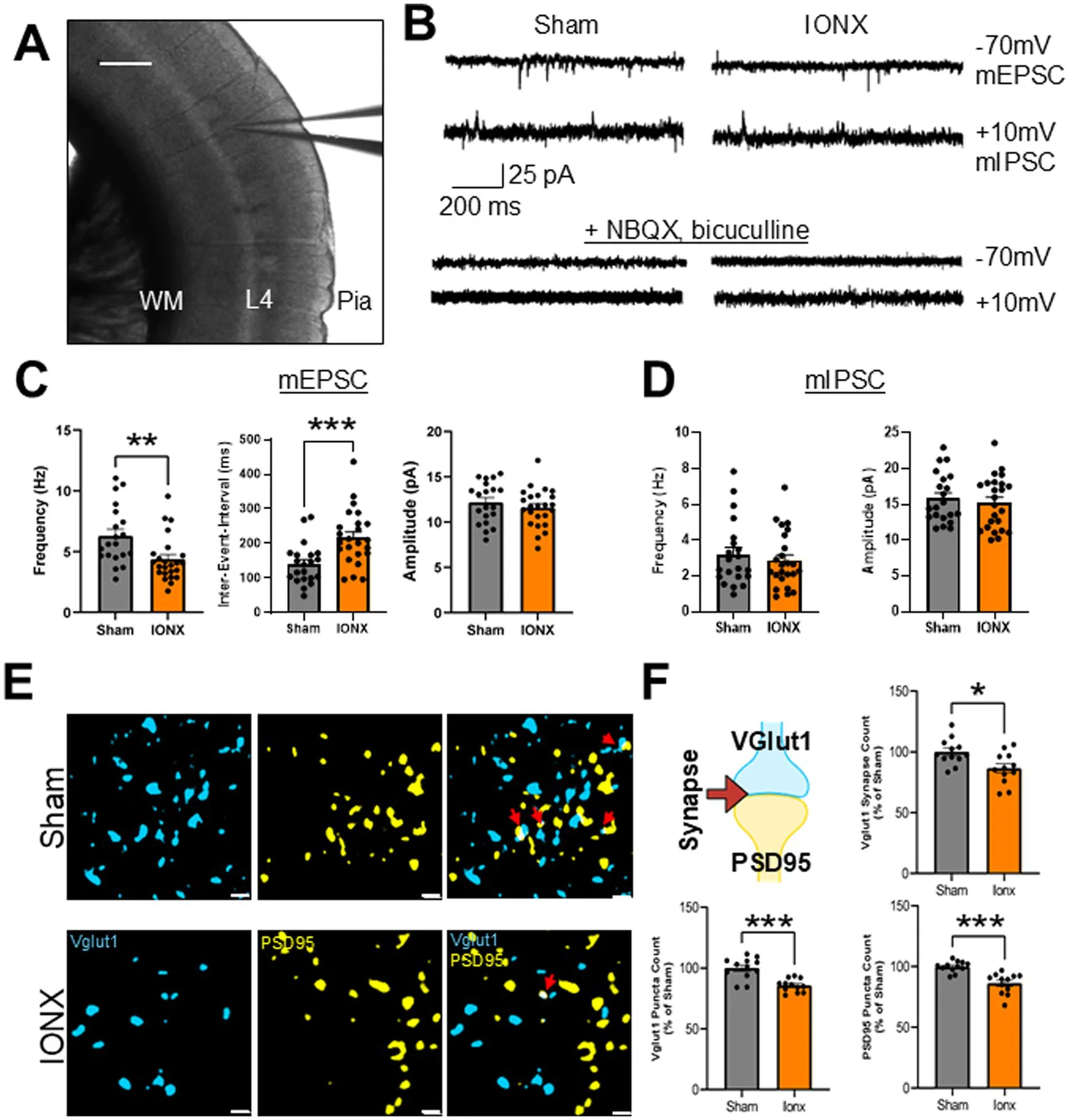
Whisker denervation globally reduces excitatory synaptic activity and putative intracortical synapses. **(A)** Recordings occurred in layer 4 (L4) neurons in deprived S1BC; note the barrel-like structures in L4 targeted by a patch pipette. **(B)** mEPSCs and mIPSCs were recorded at −70 mV and +10 mV, respectively. NBQX and bicuculline were washed on after the recordings to validate that these events were AMPA and GABA receptor-mediated, respectively. **(C)** IONX mice had reduced mEPSC frequency compared to shams (left), with an increase in inter-event interval (center). mEPSC amplitudes were unchanged in IONX compared to sham (right). **(D)** mIPSC frequency and amplitudes were not significantly different between sham and IONX. **(E)** Synapse quantification analysis identified intracortical presynaptic (VGlut1, left), postsynaptic (PSD95, center) components, and colocalized dual-labeled putative synapses (red arrows, right). **(F)** The number of VGlut1 and PSD95 components and colocalized putative synapses was reduced 7 days after IONX compared to sham. Data are presented as mean ± SEM. **(A–E)**: *n* (cells) = 21 sham and 24 IONX. (F–G): *n* (mice) = 12 sham, 12 IONX. *T*-test. * *p* < 0.05, ** *p* < 0.01, and *** *p* < 0.001. Scale bars: **A** = 500 μm, **F** = 5 μm.

### 3.3 Microglial morphology is changed after whisker denervation

Activity-mediated synapse loss can be mediated by microglial activity, which includes the selective pruning of inactive synapses during an early postnatal sensitive period (Faust et al., 2025; Gunner et al., 2019). In this study, we quantified the prevalence and morphological states of microglia in L4 of deprived S1BC in male and female mice after unilateral sham or IONX. To do this, we immunolabeled microglia with Iba1 and used the ImageJ/FIJI plugin *MicrogliaMorphology* (Kim et al., 2024) to isolate, skeletonize (Figure 3A), and categorize microglia into distinct shapes (Figure 3B). Microglia were characterized by 27 different morphological parameters (dimensions) (Supplementary Figure 3A). These categories were used to assign microglia to four different morphology phenotypes: amoeboid, hypertrophic, ramified, and bipolar (Figure 3B; Supplementary Figures 3A,B). For example, microglia with high circularity scores were assigned to an “amoeboid” shape, characterized by a circular soma with low numbers or lengths of branches. Amoeboid-shaped microglia are expected to be involved in phagocytosis, which we validated by quantifying high levels of the lysosomal protein CD68 load within this amoeboid-shaped microglia population (Supplementary Figure 3C). We then compared the prevalence of these microglial phenotypes in male and female mice 7 days after sham or IONX. We found that there was no difference in the number of microglia detected (Supplementary Table 8); however, a two-way multivariate analysis of variance detected a shift in the morphology 7 days after IONX, with no sex-specific effect (MANOVA: time point *p* = 0.015, sex *p* = 0.577). To inspect which subtype of microglia was affected, we split the morphologies and compared sham vs. IONX mice, where we found an increase in the proportion of ramified microglia and a decrease in amoeboid microglia (*t*-test *p* = 0.002, *p* = 0.032, respectively, Supplementary Table 9; Figures 3C,D). The proportion of hypertrophic and bipolar microglia was not statistically different (*t*-test, *p* = 0.725, *p* = 0.992, respectively, Supplementary Figures 4A,B). When the data were split to compare male and female microglial adaptations after IONX, we again detected a more robust shift in amoeboid and ramified microglial proportions in female than male mice (Supplementary Table 10; Supplementary Figures 4C,D). These results indicate that removal of sensory afferents via IONX alters the proportion of ramified- and amoeboid-shaped microglia in deprived S1BC.

**Figure 3:**
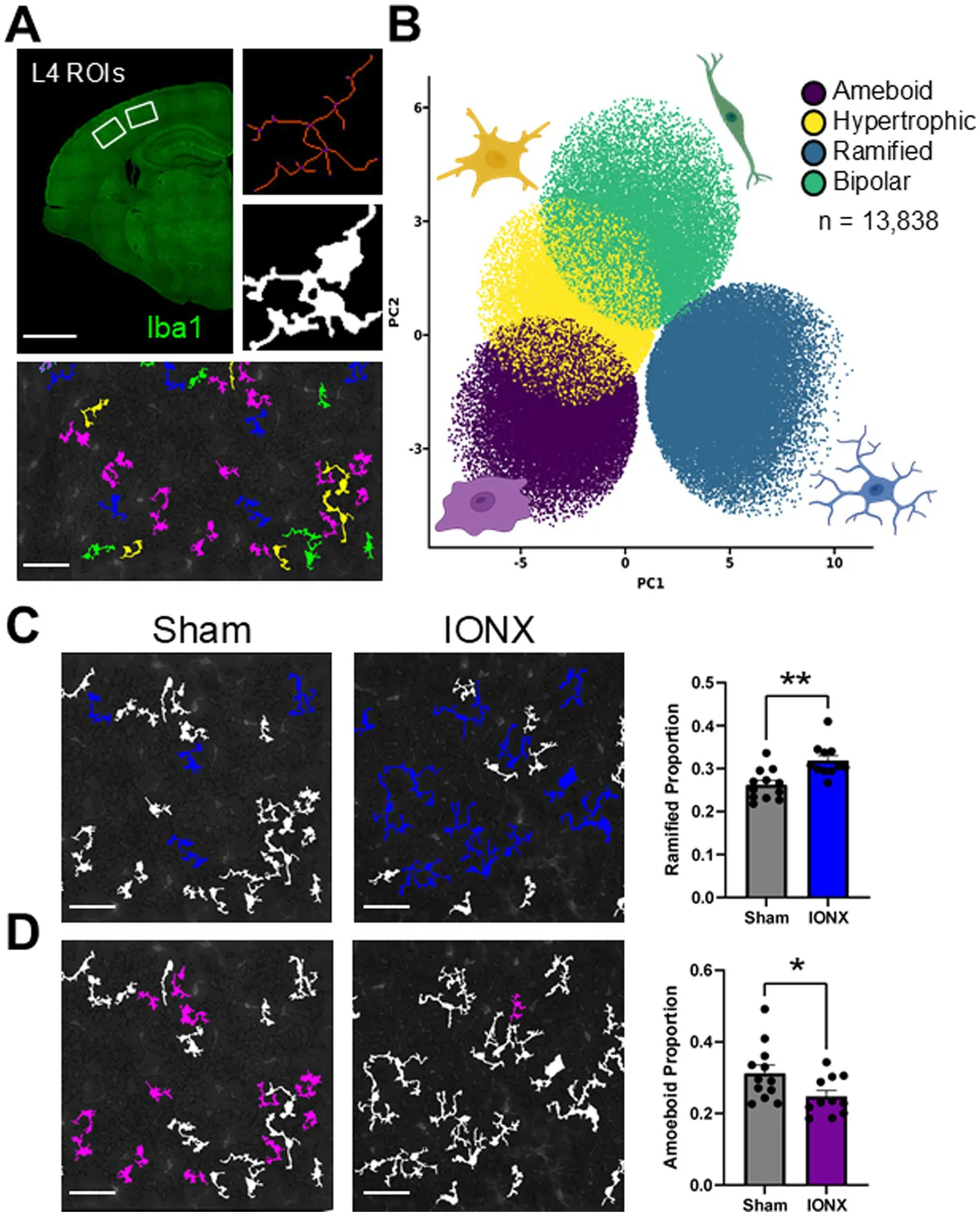
Microglial morphology is changed after whisker denervation. **(A)** Deprived S1BC containing two regions of interest (ROIs) in layer 4 (L4). Microglia are skeletonized (right) and colored (bottom) based on morphological phenotype. **(B)** Microglia are classified into four distinct phenotypes. **(C,D)** The proportion of ramified microglia increases, while the proportion of amoeboid microglia decreases 7 days after IONX compared to sham. Data are presented as mean ± SEM. *n* (mice) = 12 sham, 11 IONX. *T*-test. * *p* < 0.05 and ** *p* < 0.01. Scale bar: **A** = 1 mm, **C** and **D** = 50 μm.

### 3.4 Long-term homeostasis of deprived circuitry after whisker denervation

We detected reduced TC terminal labeling, fewer putative IC synapses, and decreased spontaneous excitatory activity in addition to altered microglial morphology in the thalamo-recipient L4 of deprived S1BC 7 days after unilateral IONX. However, most peripheral nerve injuries do not fully recover, leading to chronically dysregulated deprived cortical circuit activity. For example, thalamic atrophy and aberrant spontaneous activity are observed in deprived sensory/motor brain regions of people after limb loss (Chen et al., 1990; Draganski et al., 2006). We sought to use this model to determine whether the putative synapse loss and microglial morphology adaptations persist or worsen at chronic time points after IONX (Figure 4A). Surprisingly, 28 days after IONX VGlut2 fluorescence intensity was not different from sham mice (*t*-test *p* = 0.623, Supplementary Table 1; Figure 4B). VGlut2 fluorescence intensity did not differ in shams over time (Supplementary Figure 1C), indicating that the lack of significance was not related to aging. In addition, the proportions of ramified-shaped and amoeboid-shaped microglia were also similar between sham and IONX mice (*t*-test *p* = 0.601, 0.501, respectively, Supplementary Table 11; Figures 4C,D). Separating the data by sex did not elucidate any sex-specific adaptations at this late time point (Supplementary Table 12; Supplementary Figure 5). The same experimental procedures were performed at 14 days after sham/IONX, which determined that VGlut2 fluorescence intensity and microglial morphology were not statistically different between sham and IONX mice of either sex (Supplementary Table 1, 3, 11, 12; Supplementary Figure 6). These findings indicate that there is a transient reduction in VGlut2^+^ TC terminal labeling, corresponding with microglial morphology phenotype adaptations. Within 14 days, these circuit modifications stabilize, with the deprived S1BC reaching a stable TC innervation and microglial population.

**Figure 4:**
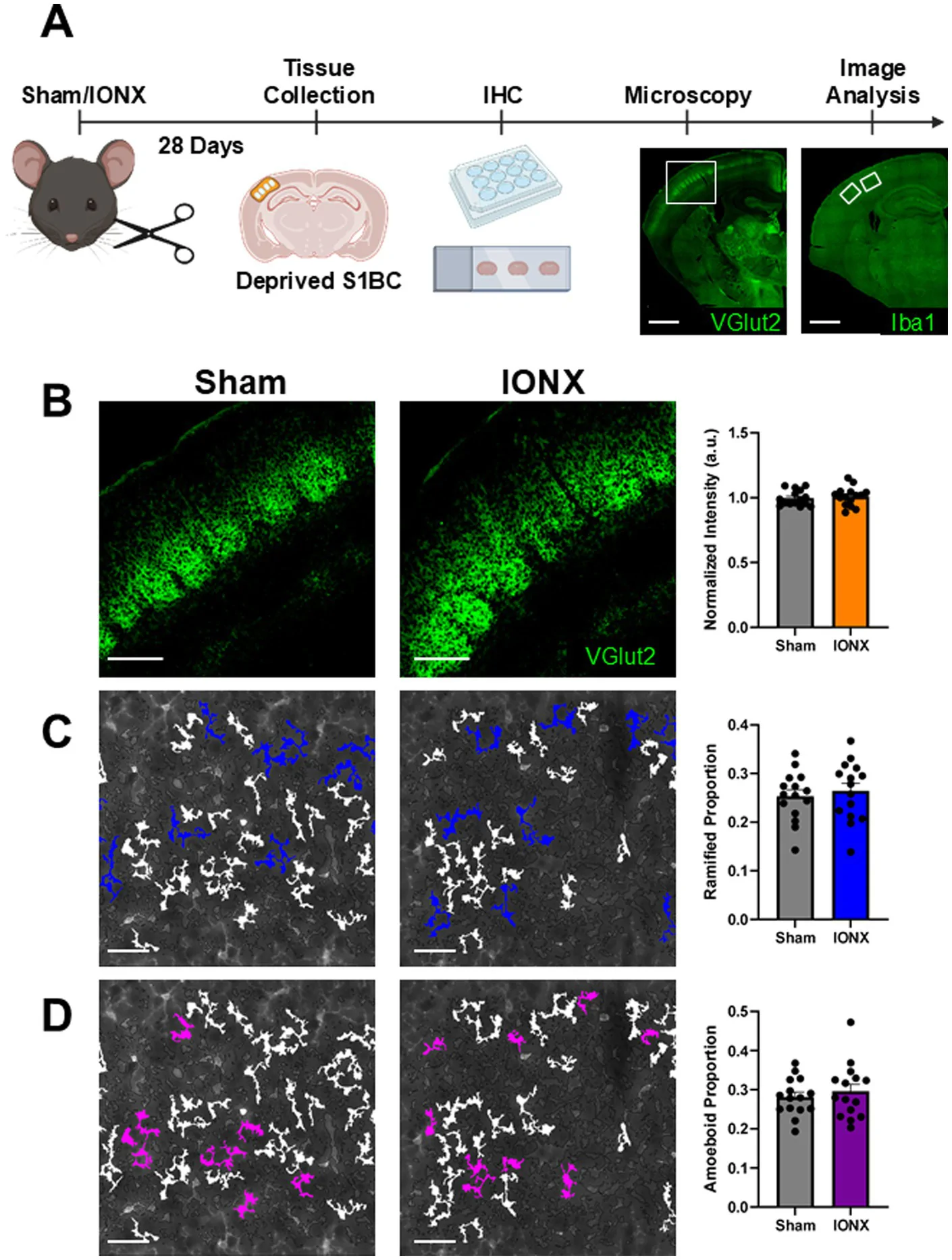
Stability of deprived circuitry after chronic whisker denervation. **(A)** Experimental timeline, immunohistochemistry (IHC) was performed 28 days after sham or IONX. ROIs from deprived S1BC were selected and analyzed for VGlut2 fluorescence intensity and microglial morphology. **(B)** VGlut2 intensity was similar between sham and IONX mice. **(C)** The prevalence of ramified-shaped and **(D)** amoeboid-shaped microglia was unchanged between sham and IONX mice. Data are presented as mean ± SEM. *n* (mice) = 15 sham, 15 IONX. Scale bars: **A**= 1 mm; **B** = 200 μm; **C** = 50 μm.

## 4 Discussion

### 4.1 Summary

The loss of peripheral sensation causes widespread and dramatic changes in cortical function (Chen and Brumberg, 2021; Sparling et al., 2024). Optimal sensory perception and recovery after denervation injury depend on neural and glial interactions to recalibrate sensory circuitry (Faust et al., 2021, 2025). While much research has focused on circuit and glial adaptations in response to sensory loss during the early postnatal sensitive period (Erzurumlu and Gaspar, 2012, 2020), the findings from adult neural and glial adaptation studies have been variable. In this study, we investigated the effect of permanent unilateral whisker denervation (IONX) on synaptic density, neural activity, and microglial activity in deprived S1BC of adult male and female mice (Figure 5A). We found that thalamocortical (TC) and intracortical (IC) excitatory synapses are lost quickly, within 7 days, corresponding with a decrease in spontaneous excitatory activity. At this time point, microglia had reduced prevalence of amoeboid-shaped microglia and increased prevalence of a ramified-shaped morphology, indicating increased surveillance of deprived S1BC (Figure 5B). Most of these effects appeared to be more pronounced in female mice, highlighting the importance of using both sexes in basic research studies (Clayton and Collins, 2014). Fourteen to 28 days after whisker denervation, terminal labeling and microglial measurements were similar to sham levels (Figure 5C), demonstrating that the sensory cortex employs a transient strategy to recalibrate circuitry to overcome this sensory loss.

**Figure 5:**
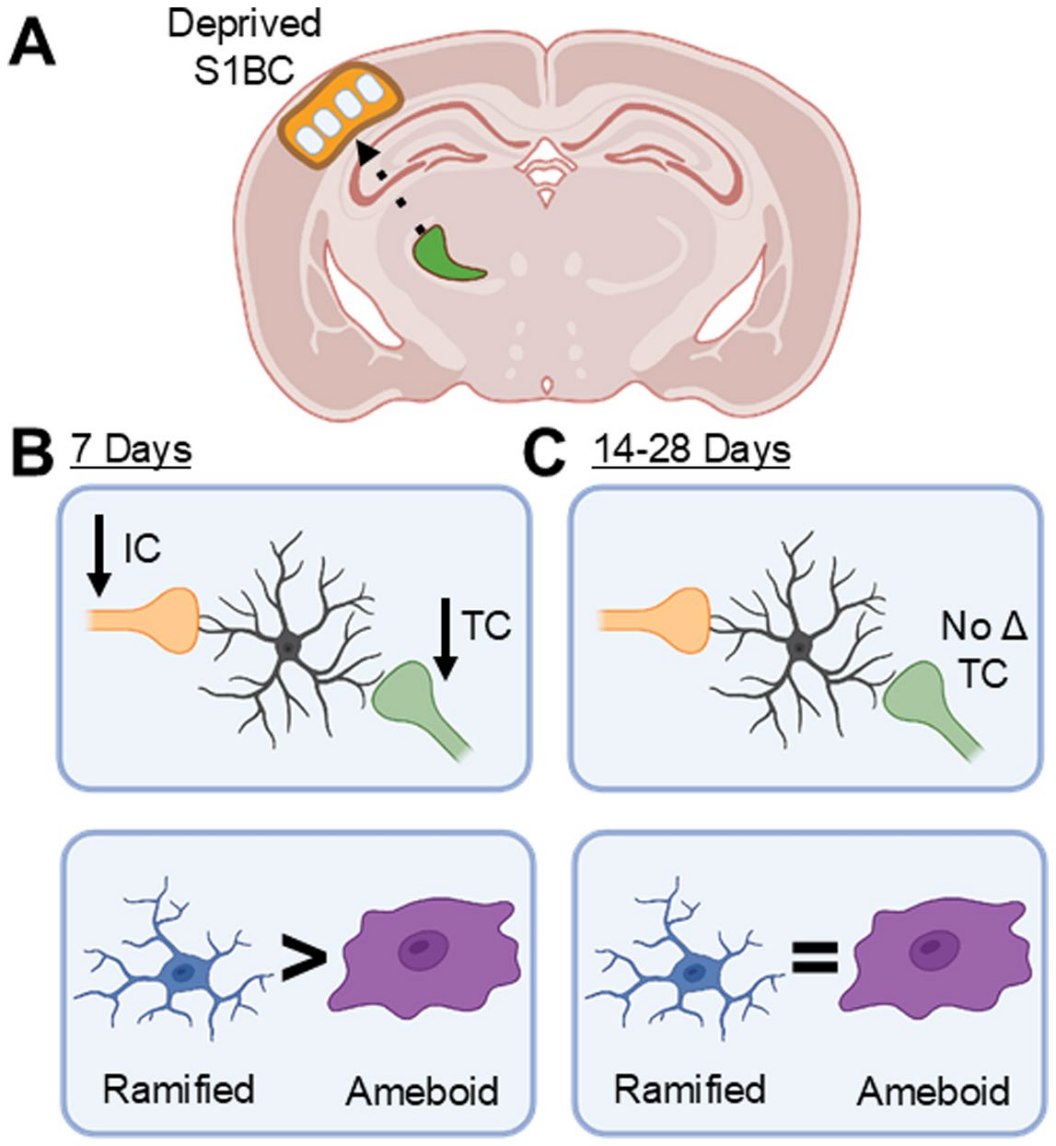
Outcome summary. **(A)** The deprived S1BC experiences a transient reduction in TC terminal labeling, putative synaptic connections, and excitatory activity. These correspond with a shift toward ramified microglial morphology 7 days after IONX **(B)**. **(C)** These measurements are stable 14–28 days after IONX.

### 4.2 Adult thalamocortical and intracortical plasticity

Seminal neuroscience experiments identified a narrow postnatal “sensitive period” of heightened sensory cortex plasticity, which uses sensory experience to refine developmental wiring into a finely tuned circuit. Closure of this sensitive period includes the stabilization of TC synapses in visual and whisker systems (Crair and Malenka, 1995; Fox, 1992; Hensch, 2005). Whisker trimming, plucking, or lesioning has long been used to manipulate circuit plasticity, during which barrels in thalamo-recipient L4 change in shape (Woolsey and Wann, 1976) and innervation density (Gunner et al., 2019; Wimmer et al., 2010) when whisker loss occurs within the first postnatal week. Although some evidence of reduced TC axon arborization was identified after whisker trimming (Oberlaender et al., 2012; Wimmer et al., 2010) in adult rodents, these deprived TC connections were largely considered aplastic.

We and others have found that sensory loss in adults can reactivate developmental-like TC plasticity mechanisms such as long-term potentiation and recruitment of silent synapses (Chung et al., 2017; Montey and Quinlan, 2011; Petrus et al., 2014; Yu et al., 2012). These modifications allow the brain to recalibrate intact neural circuitry and optimize processing of spared sensory afferents (Jie et al., 2025; Margolis et al., 2012). This potentiation of intact sensation is reminiscent of ocular dominance plasticity, where intact eye inputs are potentiated, while deprived TC inputs undergo long-term depression and synapse removal (Auguste et al., 2022; Gordon and Stryker, 1996; Smith et al., 2009; Stevens et al., 2007).

We found that the fluorescence intensity of TC terminals was only transiently reduced after IONX in adult mice (Figure 1). This raises an important question: how do deprived TC connections rebound after IONX at chronic time points? While not tested in this study, neighboring sensory territory may invade deprived S1BC to occupy this denervated cortical space. Indeed, ectopic innervation from the nearby jaw in the sensory thalamus is linked to referred hyperalgesia after IONX (Takeuchi et al., 2012, 2017; Ueta and Miyata, 2021, 2023). Subcortical reorganization has also been observed in monkeys after limb denervation (Florence et al., 2000) and in rats after paw amputation (Pearson et al., 2003; Pellicer‐Morata et al., 2024). Importantly, this subcortical reorganization occurs on a delayed timeline compared to the immediate “unmasking” of existing connections from nearby cortical areas (Pearson et al., 1999; Pellicer‐Morata et al., 2023). This resurgence of TC innervation is not restricted to the somatosensory system; for example, monocular lid suture also causes a transient reduction in TC synapses, where the delayed rebound of VGlut2 innervation to the deprived eye was hypothesized to arise from the open eye (Coleman et al., 2010).

A reduction in TC terminal labeling after IONX likely triggers a global reduction in neural activity by removing the strong afferent sensory drive. L4 neurons also lost IC putative synapses 7 days after IONX (Figure 2). L4 neurons in S1BC receive innervation from thalamic (Sermet et al., 2019; Staiger and Petersen, 2021), intracortical (Chakrabarti and Alloway, 2006; Denardo et al., 2015; Scala et al., 2019), and long-range connections (Aronoff et al., 2010). The loss of TC and IC inputs resulted in a dramatic reduction in spontaneous excitatory events (Figure 2). Although the source of mEPSC events and VGlut1^+^ puncta were not explored in this study, the reduction in excitatory activity in deprived S1BC was not restricted to TC connections. Intriguingly, despite reduced excitatory putative synapse number and activity, we did not observe altered spontaneous inhibitory events. Inhibition plays a crucial role in experience-dependent synaptic plasticity and circuit refinement (Hensch, 2005; Takesian et al., 2018). Because our experiments only explored inhibitory loss using electrophysiology, we may have missed other mechanisms by which inhibition could be altered by IONX. For example, after IONX, others found potentiated GABA_A_-receptor-mediated tonic inhibition in the thalamus (Nagumo et al., 2020). This type of inhibition is distinct from the phasic inhibition (Farrant and Nusser, 2005; Wu et al., 2013), which is usually measured with mIPSC recordings. Together, our findings identify changes in excitatory connections that occur in a temporally restricted way after whisker denervation.

### 4.3 Microglial responses to changes in neural activity

Synapse loss is often attributed to the phagocytic activity of microglia. These glia use complex signaling to identify and remove underused synapses, while leaving active synapses untouched (Faust et al., 2021, 2025; Gunner et al., 2019; Schafer et al., 2012). Microglia work with astrocytes (Faust et al., 2025) and oligodendrocyte precursor cells (Auguste et al., 2022) to mediate synapse removal after loss of sensory afferents. There are many ways to characterize microglial phenotypes, including but not limited to morphological, proteomic, and transcriptomic methods (Colombo et al., 2022; Paolicelli et al., 2022). We chose to use an automated pipeline to unbiasedly characterize microglia by morphology (Kim et al., 2024), where we found a transient shift to ramified phenotypes 7 days after IONX, which returned to sham levels by days 14–28 after injury (Figures 3, 4). Microglia with a ramified morphology are thought to be in a “surveillance” state, sampling nearby synapses to gauge network needs. Indeed, microglia are continually in this state, contacting synapses (Tremblay et al., 2010; Wake et al., 2009), supporting axon function (Umpierre et al., 2020), and increasing neural synchrony (Akiyoshi et al., 2018).

Because we found reduced TC terminal labeling and IC putative synaptic connections after IONX, we expected an increase in amoeboid, presumably “phagocytic” microglial morphology. We instead found decreased amoeboid-shaped microglia. We hypothesize that the phagocytic activity of microglia may already be complete by this 7-day time point, as is seen in younger mice (Faust et al., 2025; Gunner et al., 2019). Instead, we may have detected microglia which could be actively surveilling and helping to recalibrate deprived S1BC circuitry after IONX. Microglia are known to contact active axons (Fujita et al., 2019) and support both synaptogenesis (Miyamoto et al., 2016) and structural remodeling from ectopic TC regions (Ueta and Miyata, 2021). Fourteen days post-IONX, a new circuit activity homeostasis may have been achieved, thus restoring microglial morphology measurements to sham levels. Finally, an alternative explanation may be that microglia are not involved in pruning this TC connection in adults, which may have been accomplished by other glia such as oligodendrocyte precursor cells or astrocytes (Auguste et al., 2022; Chung et al., 2013). We found that microglia undergo a transient shift to ramified morphology; however, in the absence of additional measurements of microglial function or inclusion of more time points after injury, the role of this transiently appearing class of ramified-shaped microglia is unknown.

### 4.4 Sex-specific adaptations to sensory loss

Male and female animals and humans have sexually dimorphic responses to experience, leaving females potentially more vulnerable to negative consequences such as PTSD (Hiscox et al., 2023). This sex-specific response may occur due to different plasticity mechanisms (Copenhaver and LeGates, 2024; Gall et al., 2023), synaptic structures (Hyer et al., 2018), or spine densities (Gould et al., 1990; Woolley et al., 1990). These factors influence connectivity after stressful experiences (Honeycutt et al., 2020), which may be regulated by altered expression of the cadherins that link synapses together (Liao et al., 2024). Although the mechanisms causing the more pronounced female TC terminal labeling loss and spontaneous activity reduction after IONX (Supplementary Figures 1, 2, 4) were not explored here, microglia have sex-specific phenotypes and injury response characteristics that may impact circuit remodeling. For example, microglia in adult female rodents have increased amoeboid-shaped morphology (Schwarz et al., 2012) and increased phagocytic behavior (Yanguas-Casás et al., 2020) are neuroprotective after stroke (Villa et al., 2018). Furthermore, microglia-associated genes have been detected specifically in female rats after IONX (Orczyk et al., 2016). Female microglia may be more “alert” and primed to respond after injury (Lynch, 2022); this may create a more permissive environment for circuit remodeling in female than male mice. We conclude that some variability in whisker-deprivation circuit remodeling studies may be due to sex-specific sensory manipulation responses. For example, microglia may be more likely to phagocytize underused synapses in female than male mice.

### 4.5 Circuit remodeling and implications for recovery after sensory loss

Circuit plasticity and microglial responses to whisker denervation may have important implications for supporting recovery after denervation injury (Bayona et al., 2005; Bhat et al., 2017; Meyers et al., 2019) or limb loss (Sparling et al., 2024). Circuit remodeling after limb loss is detected as altered neural activity in the deprived sensory cortex and has been linked to phantom limb pain (Flor et al., 2006; Valyear et al., 2020). Although others find persistent representations of the phantom in the deprived cortex (Makin et al., 2013; Schone et al., 2025), these alterations in activity and connectivity (Bramati et al., 2019; Simões et al., 2012) likely occur in humans and mice via similar mechanisms. If successful recovery requires learning-induced circuit remodeling, pharmacological or therapeutic interventions should be implemented during a transient window such as the one we identified in our IONX model to capitalize on this cortical plasticity. If circuit remodeling instead underlies problematic adaptations such as the establishment of phantom limb pain, preventing circuit remodeling may prevent this outcome. Indeed, microglial depletion in this IONX model prevented referred hyperalgesia (Ueta and Miyata, 2021) and has also shown benefit for stroke recovery (Isla Cainzos et al., 2026). Although it is still debated whether circuit remodeling after denervation injury or limb loss is beneficial or problematic, our findings indicate that temporal and sex-specific adaptations may play important roles in how the brain responds to peripheral injury.

## Data Availability

The raw data supporting the conclusions of this article will be made available by the authors, without undue reservation.

## Footnotes

1
https://www.protocols.io/view/synbot-protocols-3byl4qewjvo5/v2

## Supplementary material

SUPPLEMENTARY FIGURE 1 TC Terminal Labeling Split by Sex and Timepoint. **(A,B)** VGlut2 fluorescence intensity reduction appears to be more robust in female than male mice 7 days after IONX. n (mice) = 6M sham, 8F sham, 4M IONX, 8F IONX. **(C)** VGlut2 intensity does not differ between days 7, 28 in sham mice n (mice) = 14 sham, 15 IONX. No sex specific changes were detected between sham male or female mice. Data is presented as mean ± SEM. *T*-test **p* < 0.05.

SUPPLEMENTARY FIGURE 2 Activity and Putative IC Synapse Reduction 7 days after IONX appears to be more robust in female mice than male mice. **(A)** Male mEPSC frequency trends downward, inter-event-interval (IEI) and amplitudes are unchanged after IONX. **(B)** Female mEPSC frequency is significantly decreased, IEI is significantly increased, and amplitudes are unchanged. **(C)** mIPSC frequency and amplitudes are unchanged in male (left) or female (right) mice 7 days after IONX compared to shams. **(D)** VGlut1 (left) and PSD95 (center) are significantly decreased in male and female mice, but putative synapse (right) loss is only significant in female mice 7 days after IONX. Data is presented as mean ± SEM. **A–C**: n (cells) = 12M sham, 9F sham, 12M IONX, 12F IONX. **D**: n (mice) = 6M sham, 6F sham, 6M IONX, 6F IONX. *T*-test ^#^*p* < 0.1, **p* < 0.05, ***p* < 0.01.

SUPPLEMENTARY FIGURE 3 Microglia Morphology Phenotype Detection Parameters and Validation. **(A)** 27 morphological parameters were used to categorize microglia into 4 subtypes. **(B)** The ideal number of clusters was determined to be 4, as detected by k-means clustering and data dimensions (inset). **(C)** Ameboid microglia were determined to have a high CD68 load, while ramified microglia had low CD68 levels.

SUPPLEMENTARY FIGURE 4 Microglial Phenotypes 7 Days after Sham or IONX. **(A,B)** Hypertrophic and bipolar microglia have similar proportions in sham and IONX mice. **(C,D)** Male mice have only a trend of increased ramified phenotypes after IONX, while females are significantly upregulated. Data is presented as mean ± SEM. n (mice) = 5M sham, 7F sham, 4M IONX, 7F IONX. *T*-test ^#^*p* < 0.1, **p* < 0.05.

SUPPLEMENTARY FIGURE 5 Microglial Phenotypes 28 Days after Sham or IONX. **(A,B)** Hypertrophic and bipolar microglia have similar proportions in sham and IONX mice. **(C,D)** No sex-specific changes in microglial morphology were detected. Data is presented as mean ± SEM. **A,B**: n (mice) = 8M sham, 7F sham, 8M IONX, 7F IONX.

SUPPLEMENTARY FIGURE 6 No changes in VGlut2^+^ TC terminal fluorescence intensity or microglial morphology 14 days after IONX compared to sham. **(A)** Experimental timeline, immunohistochemistry (IHC) was performed 14 days after sham or IONX. **(B)** VGlut2 fluorescence intensity is similar between sham and IONX mice. **(C)** Microglial morphology is similar between sham and IONX mice. **(D)** No sex-specific adaptations in microglial morphology were observed. Data is presented as mean ± SEM. **B**: n (mice) = 12 sham, 13 IONX. **C**: n (mice) = 11 sham, 13 IONX. **D**: n (mice) = 6M sham, 5F sham, 7M IONX, 6F IONX.
