# Supplementary material for: Transient synaptic refinement and glial adaptations after whisker denervation in adult mice
Source: Front Cell Neurosci. 2026 Sep 30;20:1941129. doi: 10.3389/fncel.2026.1941129 (PMC13662750; doi:10.3389/fncel.2026.1941129)
Supplement: Supplementary file 1 [file Table_1.docx]

Supplemental Table 1: Values for Figure 1, 4, Supplemental Figure 6.

| **VGlut2 Fluorescence Intensity** | **Sham** | **IONX** | **TTEST**  **Sham vs IONX** |
| --- | --- | --- | --- |
| 7D | (14)  1 ± 0.057 | (12)  0.921 ± 0.063 | **0.003** |
| 14D | (12)  1 ± 0.063 | (13)  0.988 ± 0.055 | 0.610 |
| 28D | (15)  0.997 ± 0.059 | (15)  1.01 ± 0.072 | 0.623 |

Data listed as (Number of Animals) normalized mean ± SEM. Students T-tests were performed on sham vs. IONX in male and female cohorts separately. **Bold** indicates statistical significance (p<0.05) and *italics* indicates trend (0.05<p<0.1).

Supplemental Table 2: Values for Figure 1

| **VGlut2 Fluorescence Intensity** | **M Sham** | **F Sham** | **TTEST**  **M Sham vs F Sham** |
| --- | --- | --- | --- |
| 7D | (6)  1340 ± 218.2 | (8)  1376 ± 273.0 | 0.791 |
| 28D | (8)  1160 ± 197.9 | (7)  1277 ± 297.2 | 0.398 |

Data listed as (Number of Animals) normalized mean ± SEM. Students T-tests were performed on sham vs. IONX in male and female cohorts separately. **Bold** indicates statistical significance (p<0.05) and *italics* indicates trend (0.05<p<0.1).

Supplemental Table 3: Values for Figure 1, 4, Supplemental Figures 1, 6.

| **VGlut2 Fluorescence Intensity** | **M Sham** | **M IONX** | **F Sham** | **F IONX** | **TTEST**  **Sham vs IONX** |
| --- | --- | --- | --- | --- | --- |
| 7D | (6)  1 ± 0.038 | (4)  0.939 ± 0.027 | (8)  1 ± 0.019 | (8)  0.911 ± 0.018 | Male: 0.282  **Female:0.005** |
| 14D | (7)  1 ± 0.026 | (7)  0.979 ± 0.043 | (5)  1 ± 0.029 | (6)  0.998 ± 0.029 | Male: 0.491  Female: 0.963 |
| 28D | (8)  0.994 ± 0.018 | (8)  1 ± 0.024 | (7)  1 ± 0.027 | (7)  1.019 ± 0.030 | Male: 0.858  Female: 0.648 |

Data listed as (Number of Animals) normalized mean ± SEM. Students T-tests were performed on sham vs. IONX in male and female cohorts separately. **Bold** indicates statistical significance (p<0.05) and *italics* indicates trend (0.05<p<0.1).

Supplemental Table 4: 3-way ANOVA for data in Figures 1, 4, Supplemental Figure 6.

| All | DF | Sum Sq | Mean Sq | F value | p val |
| --- | --- | --- | --- | --- | --- |
| **Treatment** | 1 | 1.10E-02 | 1.10E-02 | 2.71658787 | 0.10385662 |
| **Timepoint** | 2 | 2.46E-02 | 1.23E-02 | 3.02330806 | *0.05512774* |
| **Sex** | 1 | 1.26E-04 | 1.26E-04 | 0.03094665 | 0.86087474 |
| **Treatment:Timepoint** | 2 | 3.06E-02 | 1.53E-02 | 3.76268316 | **0.02811944** |
| **Treatment:Sex** | 1 | 7.74E-05 | 7.74E-05 | 0.01903216 | 0.89067615 |
| **Timepoint:Sex** | 2 | 2.29E-03 | 1.14E-03 | 0.28129316 | 0.75566842 |
| **Treatment:Timepoint:Sex** | 2 | 2.01E-03 | 1.01E-03 | 0.24724801 | 0.78163589 |
| **Residuals** | 69 | 2.81E-01 | 4.07E-03 | NA | NA |

3-way ANOVA was run on the normalized VGlut2 intensity of all samples (Treatment x Time Point x Sex). **Bold** indicates statistical significance (p<0.05) and *italics* indicates trend (0.05<p<0.1).

Supplemental Table 5: mEPSC Parameters for Figure 2C

| **mEPSC** | **M Sham**  **(12, 4)** | **M IONX**  **(12, 4)** | **F Sham**  **(9, 3)** | **F IONX**  **(12, 4)** | **TTEST**  **Sham vs IONX** |
| --- | --- | --- | --- | --- | --- |
| Frequency (Hz) | 6.2 ±0.7 | 4.5 ± 0.5 | 6.5 ± 0.8 | 4.2 ± 0.6 | Male: 0.082  **Female:0.041** |
| Amplitude (pA) | 11.6 ± 0.6 | 11.3 ± 0.5 | 13.1 ± 0.9 | 11.9 ± 0.7 | Male: 0.746  Female: 0.284 |
| Rise Time (ms) | 0.9 ± 0.2 | 0.9 ± 0.1 | 0.9 ± 0.1 | 1.0 ± 0.1 | Male: 0.969  Female: 0.286 |
| Decay (ms) | 2.1 ± 0.2 | 2.1 ± 0.2 | 2.6 ± 0.3 | 2.9 ± 0.2 | Male: 0.933  Female: 0.541 |
| Area (fC) | 16.1 ± 1.7 | 14.7 ± 0.8 | 19.9 ± 1.4 | 17.5 ± 0.7 | Male: 0.457  Female: 0.150 |
| Series R (MΩ) | 23.2 ± 1.2 | 26 ± 0.7 | 25 ± 0.7 | 25 ± 1 | Male: 0.070  Female: 0.956 |
| Input R (MΩ) | 114 ± 3.8 | 130 ± 7 | 127 ± 11 | 140 ± 15 | Male: 0.077  Female: 0.474 |

Values are Average ± Standard Error of the Mean (SEM). Animal n = (cell, mouse).

Students T-tests were performed on sham vs. IONX in male and female cohorts separately. Male sham vs Female sham are not statistically different (p>0.05).

Male IONX vs Female IONX are not statistically different except for Area (p = 0.03) and Decay (p = 0.02).

Supplemental Table 6: mIPSC Parameters for Figure 2D

| **mIPSC** | **M Sham (12, 4)** | **M IONX**  **(12, 4)** | **F Sham**  **(9, 3)** | **F IONX**  **(12, 4)** | **TTEST**  **Sham vs IONX** |
| --- | --- | --- | --- | --- | --- |
| Frequency (Hz) | 2.7 ± 0.34 | 3.1 ± 0.4 | 3.9 ± 0.8 | 2.7 ± 0.5 | Male: 0.467  Female:0.210 |
| Amplitude (pA) | 16.6 ± 1.0 | 15.3 ± 0.9 | 14.9 ± 1.1 | 15.2 ± 1.3 | Male: 0.337  Female: 0.842 |
| Rise Time (ms) | 2.3 ± 0.3 | 2.3 ± 0.2 | 2.3 ± 0.3 | 2.1 ± 0.2 | Male: 0.893  Female: 0.633 |
| Decay (ms) | 7.4 ± 0.8 | 6.9 ± 0.4 | 6.4 ± 0.9 | 7.4 ± 0.5 | Male: 0.541  Female: 0.370 |
| Area (fC) | 84.3 ± 14 | 61.4 ± 3.9 | 62.8 ± 15 | 66.4 ± 9.9 | Male: 0.139  Female: 0.838 |
| Series R (MΩ) | 23.2 ± 1.2 | 26 ± 0.7 | 25 ± 0.7 | 25 ± 1 | Male: 0.070  Female: 0.956 |
| Input R (MΩ) | 114 ± 3.8 | 130 ± 7 | 127 ± 11 | 140 ± 15 | Male: 0.077  Female: 0.474 |

All values are Average ± Standard Error of the Mean (SEM). Animal n = (cell, mouse).

Students T-tests were performed on sham vs. IONX in male and female cohorts separately. Male sham vs Female sham are not statistically different (p>0.05).

Male IONX vs Female IONX are not statistically different (p>0.05).

Supplemental Table 7: Putative synapse quantification. Values for Figure 2F

| **VGlut1 Synapse** | **M Sham (6)** | **M IONX (6)** | **F Sham (6)** | **F IONX (6)** | **TTEST**  **Sham vs IONX** |
| --- | --- | --- | --- | --- | --- |
| VGlut1 | 100 ± 3.871 | 87.09 ± 2.158 | 100 ± 3.756 | 84.51 ± 2.218 | **Male: 0.0199,**  **Female:0.0073**  **Cohen’s D: 2.44** |
| PSD95 | 100 ± 1.670 | 84.30 ± 3.890 | 100 ± 2.084 | 88.31 ± 3.084 | **Male: 0.008**  **Female: 0.0123**  **Cohen’s D: 2.24** |
| Putative Synapse | 100 ± 2.955 | 92.10 ± 3.289 | 100 ± 5.859 | 80.92 ± 6.232 | Male: 0.1048  **Female: 0.049**  **Cohen’s D: 1.19** |

Data listed as (number of mice) normalized mean ± SEM. Students T-tests were performed on sham vs. IONX in male and female cohorts separately. **Bold** indicates statistical significance (p<0.05) and *italics* indicates trend (0.05<p<0.1).

Supplemental Table 8: Number of microglia per ROI in L4. Values for Figures 3, 4, Supplemental Figures 4-6

| **Density** | **M Sham** | **M IONX** | **F Sham** | **F IONX** | **TTEST**  **Sham vs IONX** |
| --- | --- | --- | --- | --- | --- |
| 7D | (5)  17.58 ± 2.229 | (4)  19.48 ± 2.565 | (7)  19.64 ± 1.412 | (7)  19.49 ± 1.735 | Male: 0.594  Female:0.948 |
| 14D | (6)  23.26 ± 1.838 | (7)  21.85 ± 1.511 | (5)  18.57 ± 2.668 | (6)  16.26 ± 1.827 | Male: 0.566  Female: 0.497 |
| 28D | (8)  21.88 ± 1.264 | (8)  22.34 ± 1.562 | (7)  22.93 ± 1.138 | (7)  20.32 ± 1.718 | Male: 0.818  Female: 0.233 |

Data listed as (n = mice), mean density ± SEM. Students T-tests were performed on sham vs. IONX in male and female cohorts separately.

Supplemental Table 9: Microglia morphology values for Figure 3

| **Morphology (7D)** | **Sham (1860, 12)** | **IONX (1605, 11)** | **TTEST: Sham vs IONX** |
| --- | --- | --- | --- |
| Amoeboid | 0.312 ± 0.023 | 0.238 ± 0.053 | **0.0323** |
| Hypertrophic | 0.215 ± 0.048 | 0.224 ± 0.064 | 0.725 |
| Ramified | 0.262 ± 0.035 | 0.318 ± 0.038 | **0.002** |
| Bipolar | 0.210 ± 0.045 | 0.210 ± 0.031 | 0.992 |

Data listed as (Number of cells, mice) mean proportion ± SEM. Students T-tests were performed on sham vs. IONX in male and female cohorts separately. **Bold** indicates statistical significance (p<0.05) and *italics* indicates trend (0.05<p<0.1).

Supplemental Table 10: Microglia morphology values for Supplemental Figure 4

| **Morphology (7D)** | **M Sham (760, 5)** | **M IONX**  **(674, 4)** | **F Sham**  **(1100, 7)** | **F IONX**  **(931, 7)** | **TTEST**  **Sham vs IONX** |
| --- | --- | --- | --- | --- | --- |
| Amoeboid | 0.294 ± 0.038 | 0.266 ± 0.027 | 0.326 ± 0.029 | 0.260 ± 0.020 | Male: 0.197  *Female:0.094* |
| Hypertrophic | 0.203 ± 0.028 | 0.244 ± 0.036 | 0.224 ± 0.015 | 0.213 ± 0.023 | Male: 0.402  Female: 0.684 |
| Ramified | 0.264 ± 0.020 | 0.337 ± 0.026 | 0.261 ± 0.012 | 0.308 ± 0.010 | *Male: 0.066*  **Female: 0.011** |
| Bipolar | 0.239 ± 0.014 | 0.193 ± 0.022 | 0.189 ± 0.016 | 0.219 ± 0.007 | Male: 0.137  Female: 0.121 |

Data listed as (Number of cells, mice) mean proportion ± SEM. Students T-tests were performed on sham vs. IONX in male and female cohorts separately. **Bold** indicates statistical significance (p<0.05) and *italics* indicates trend (0.05<p<0.1). Male sham vs Female sham are only statistically different (p<0.05) for bipolar proportion at 7D.

Supplemental Table 11: Microglia morphology values for Figure 4

| **Morphology (28D)** | **Sham (2593, 15)** | **IONX (2567, 15)** | **TTEST: Sham vs IONX** |
| --- | --- | --- | --- |
| Amoeboid | 0.281 ± 0.048 | 0.296 ± 0.067 | 0.501 |
| Hypertrophic | 0.249 ± 0.067 | 0.232 ± 0.061 | 0.475 |
| Ramified | 0.254 ± 0.051 | 0.264 ± 0.061 | 0.601 |
| Bipolar | 0.216 ± 0.033 | 0.210 ± 0.035 | 0.480 |

Data listed as (Number of cells, mice) mean proportion ± SEM. Students T-tests were performed on sham vs. IONX in male and female cohorts separately. **Bold** indicates statistical significance (p<0.05) and *italics* indicates trend (0.05<p<0.1).

Supplemental Table 12: Microglia morphology values for Supplemental Figure 5

| **Morphology (28D)** | **M Sham (1309, 8)** | **M IONX**  **(1429, 8)** | **F Sham**  **(1284, 7)** | **F IONX**  **(1138, 7)** | **TTEST**  **Sham vs IONX** |
| --- | --- | --- | --- | --- | --- |
| Amoeboid | 0.272 ± 0.017 | 0.273 ± 0.017 | 0.292 ± 0.017 | 0.322 ± 0.032 | Male: 0.951  Female:0.432 |
| Hypertrophic | 0.268 ± 0.024 | 0.244 ± 0.022 | 0.227 ± 0.028 | 0.219 ± 0.023 | Male: 0.467  Female: 0.805 |
| Ramified | 0.240 ± 0.023 | 0.289 ± 0.018 | 0.269 ± 0.011 | 0.236 ± 0.023 | Male: 0.116  Female: 0.241 |
| Bipolar | 0.219 ± 0.014 | 0.193 ± 0.011 | 0.212 ± 0.010 | 0223 ± 0.013 | Male: 0.171  Female: 0.523 |

Data listed as (Number of cells, mice) mean proportion ± SEM. Students T-tests were performed on sham vs. IONX in male and female cohorts separately. **Bold** indicates statistical significance (p<0.05) and *italics* indicates trend (0.05<p<0.1). Male sham vs Female sham are only statistically different (p<0.05) for bipolar proportion at 7D.
